# INTERGENERATIONAL TRANSFERS, RESILIENCE, AND SELF-RATED HEALTH: FINDINGS FROM HONOLULU AND WUHAN

**DOI:** 10.1093/geroni/igz038.2190

**Published:** 2019-11-08

**Authors:** Sizhe Liu, Wei Zhang, Keqing Zhang, Bei Wu

**Affiliations:** 1 University of Hawaii at Manoa, Honolulu, Hawaii, United States; 2 New York University, New York, New York, United States

## Abstract

This study investigated the association between intergenerational transfer and self-rated health (SRH) using data collected from 323 older Chinese immigrants in Honolulu and 752 Chinese older adults in Wuhan. We also explored the mediating role of resilience and the moderating role of gender in linking the associations. Findings show, for both study sites, receiving greater emotional support was associated with increased levels of SRH and this association was partially explained by resilience for females but not for males. For country differences, receiving economic support was negatively associated with SRH whereas providing economic support was positively associated with SRH for males but not for females in Honolulu. In Wuhan, receiving housework support was negatively associated with SRH for females but not for males. These findings indicate that future studies need to consider country differences as well as the impact of acculturation when examining intergenerational transfers and health among Chinese older adults.

